# Role of Microglia in Oxidative Toxicity Associated with Encephalomycarditis Virus Infection in the Central Nervous System

**DOI:** 10.3390/ijms13067365

**Published:** 2012-06-14

**Authors:** Yasuhisa Ano, Akikazu Sakudo, Takashi Onodera

**Affiliations:** 1 Central Laboratories for Frontier Technology, Kirin Holdings Co, Ltd, 1–13-5 Fukuura Kanazawa-ku, Yokohama-shi, Kanagawa 236-0004, Japan; E-Mail: Yasuhisa_Ano@kirin.co.jp; 2 Laboratory of Biometabolic Chemistry, School of Health Sciences, Faculty of Medicine, University of the Ryukyus, 207 Uehara, Nishihara, Okinawa 903-0215, Japan; E-Mail: sakudo@med.u-ryukyu.ac.jp; 3 Graduate School of Agricultural and Life Sciences, The University of Tokyo, 1-1-1 Yayoi, Bunkyo-ku, Tokyo 113-8657, Japan

**Keywords:** encephalomyocarditis virus (EMCV), reactive oxygen species (ROS), NADPH oxidase (NOX), microglia

## Abstract

The single-stranded RNA encephalomyocarditis virus (EMCV) can replicate in the central nervous system (CNS) and lead to prominent brain lesions in the stratum pyramidale hippocampus and the stratum granulosum cerebelli. Activated microglia cells infected by EMCV produce a massive burst of reactive oxygen species (ROS) via NADPH oxidase 2 (NOX2) activation, leading to neuronal death. Balancing this effect is mechanisms by which ROS are eliminated from the CNS. Cellular prion protein (PrP^C^) plays an important antioxidant role and contributes to cellular defense against EMCV infection. This review introduces recent knowledge on brain injury induced by EMCV infection via ROS generation as well as the involvement of various mediators and regulators in the pathogenesis.

## 1. Introduction

In the central nervous system (CNS), reactive oxygen species (ROS), including superoxide and hydrogen peroxide, are generated as toxic byproducts in metabolic processes. Under normal physiological conditions, regulated ROS generation plays an important role in diverse vital functions including host defense (e.g., antiviral and antibacterial effects), oxygen sensing, and signal transduction [1,2]. However, overproduction of ROS due to exaggerated innate immune responses can be toxic to host cells such as neurons in the CNS [3,4]. The relationship between viral infection of the CNS and ROS-mediated injury of neuronal cells has gradually been elucidated. The single-stranded RNA encephalomyocarditis virus (EMCV) is a cardiovirus of the family picornaviridae [5]. Inoculation of mice with EMCV B variant (EMCV-B) via the intracranial route induces brain inflammation, and this model has been used to study brain inflammation under experimental conditions [6]. It is recently reported that the excessive ROS produced in microglia by EMCV-B infection are severely toxic for neuronal cells in mouse brains [7]. ROS are considered to be closely involved in inflammation and neuronal toxicity in CNS. Among the ROS generating enzymes, NADPH oxidases (NOX) are the major source of ROS. NOX was first identified in phagocytes such as macrophage and microglia [8].

Microglial cells are distributed throughout the central nervous system as a network of immunocompetent cells derived from the monocyte/macrophage lineage. Microglial cells are activated in response to cerebral ischemia, infection, and neurodegenerative diseases and form the bulk of the response to endotoxin lipopolysaccharides (LPS) in the central nervous system (CNS) [9,10]. LPS-activated microglia release inflammatory mediators, such as ROS, nitric oxide (NO), and prostaglandins. Multiple signaling molecules are activated in response to LPS in microglia. Microglia are also activated by viral infection of the CNS and generate massive amounts of cytotoxic ROS via NOX2, which in turn induce neuronal apoptosis. This review focuses on the role of innate immunity against EMCV infection and the mechanisms of ROS-mediated cytotoxicity in CNS neurons under conditions of EMCV infection.

## 2. Encephalomycarditis Virus

Encephalomycarditis virus (EMCV) is a cardiovirus that belongs to the family Picornaviridae [5]. This virus was first isolated from non-human primates in 1945 [11]. Small rodents, especially rats, have been suspected to be reservoir hosts or carriers for the virus [12]. EMCV has a wide host range among domestic and wild animals, and it has a worldwide distribution [13,14]. EMCV was initially classified according to 2 types; *i.e.*, type E and type M [15]. The type E variant induces a rapidly fatal infection and the type M variant induces non-fatal disease in mice. In 1980, Yoon *et al.* performed repeat plaque purification of the M variant and established a highly diabetogenic variant in mice, classified as type D, and a non-diabetogenic variant, defined as type B [16].

Kilham *et al.* examined the intracerebral route of EMCV infection in 1955. It was reported that albino rats infected intracerebrally with any of 3 different strains of EMCV-M isolated from primates developed degenerative changes and inflammatory cell infiltration in the brain, spinal cord and muscles [17]. Syrian hamsters showed viral replication in the brain and small focal areas of necrosis of neurons in the stratum pyramidal hippocampus and the stratum granulose cerebella following EMCV-D infection [18,19]. Introduction of EMCV-B virus by the intracerebral route in mice leads to replication of the virus in the CNS, indicating that cells in the CNS have receptor sites for EMCV-B [20].

In 1988, Doi *et al.* reported that BALB/c mice inoculated by the intraperitoneal route with a high dose of EMCV-D developed prominent brain lesions in the stratum pyramidale hippocampus and the stratum granulosum cerebelli [21]. The brain lesions were characterized by degeneration of neurons containing viral antigens. Mononuclear cells infiltration, spreading to the adjacent brain tissue, and thrombosis in small vessels were frequently seen. The dominant target organs of EMCV are the CNS, pancreas, and heart. In the brain, microglia cells play a central role in host defense, acting as mononuclear cells. Recent studies have gradually elucidated the recognition mechanism of antigen-presenting cells for viruses including EMCV. As an outcome of this research, EMCV is now used as model of CNS inflammation. In the following sections, we focus on innate immunity and oxidative toxicity in EMCV infection of the CNS.

## 3. Innate Immunity in EMCV Virus Infection

It has recently been reported that antigen-presenting cells recognize EMCV virus via an MDA5-mediated mechanism. In innate immunity, host pattern recognition receptors, such as Toll-like receptors (TLRs) and helicase family members, play an essential role in the recognition of molecular patterns specific for different viruses, including viral DNA, single-stranded (ss) RNA, dsRNA, and glycoproteins [22–24]. dsRNA can be generated during viral infection as a replication intermediate for RNA viruses. TLR3, which localizes in the endosomal membrane, has been shown to recognize viral dsRNA as well as the synthetic dsRNA analogue poly(I:C) [25]. The cytoplasmic proteins RIG-I and MDA5 have been also identified as dsRNA detectors [26]. RIG-I and MDA5 contain 2 caspase-recruitment domain (CARDs) and a DExD/H-box helicase domain. RIG-I recruits a CARD-containing adaptor, IPS-1 [27]. IPS-1 relays the signal to the kinases TBK1 and IKK-I, which phosphorylate interferon regulatory factor-3 (IRF-3) and IRF-7, transcription factors essential for the expression of type-I interferons [28]. It has been reported that both RIG-I and MDA-5 can bind to poly(I:C) and respond to RNA viruses *in vitro* [29]. RIG-I–deficient mice and MAD-5–deficient mice have recently been generated and EMCV was introduced to these mice [30]. In this report, the induction of interferon-α, interferon-β and IL-6 is severely impaired in the sera of MAD-5–deficient mice but not in the RIG-1–deficient. MDA-5–deficient mice and mice null for the IFN-α/β receptor were highly susceptible to EMCV infection compared to littermate controls. In contrast, deficiency of RIG-I or TLR3 did not affect the survival of mice infected with EMCV. Thus, MDA-5–mediated recognition of EMCV is a prerequisite for triggering antiviral responses as well as for prevention of dysfunction. In the CNS, astrocytes and microglia constitutively express both the RIG-I and MDA-5 transcript and protein [31]. In addition, the expression of MDA-5 is up-regulated by vesicular stomatitis virus infection *in vivo* [32]. Thus, it appears that these molecules play important roles in the detection of CNS viruses and the initiation of protective immune responses or, alternatively, the progression of damaging inflammation in the brain.

## 4. Microglia Activation by Virus Infection in the CNS

Microglia play an important role in immunological and host-defense functions in neuronal tissues. Microglia have an antigen-presenting function and activated microglia release cytokines such as IFN-α, TNF-α and IL-1β, ROS, and NOS in order to clear the antigen. Replication of a double-stranded RNA (2 kb) derived from the intermediary body of the EMCV genome was recognized by antigen-presenting cells in the MDA5, which is distributed in the cytoplasm as a RNA sensor [22]. Microglia serve as antigen-presenting cells in the CNS; thus it is microglia that recognize EMCV invasion via MDA5. Ano *et al.* reported that microglia were activated by EMCV-B infection and produced ROS via NADPH oxidase 2 (NOX2) [7]. In Ano’s report, EMCV infection activates only NOX2 in the NOX family. NOX2 is expressed by antigen-presenting cells, macrophages in lymphoid tissues, and microglia in the CNS. Recently it is reported that NOX2 and ROS are required for the host cell to trigger an efficient RIG-I or MDA-5 mediated IRF-3 activation and downstream antiviral IFN-β and *IFIT1* gene expression [33]. ROS derived from NOX2 contributes to phagocytes to produce type I interferon. ROS generated by NOX2 clear invading pathogens; however, overproduction of ROS by NOX2 in microglia is toxic to neurons.

The NOXs are a family of membrane-bound enzymes that catalyze the NADPH-dependent reduction of O_2_ to superoxide anion (O_2_^•−^). The NOX family consists of 7 isoforms, which include NOX 1–5 and dual oxidase (DUOX) 1 and 2 [8,34,35]. There are conserved structural features common amongst all homologues, including NADPH- and FAD-binding sites, 6 transmembrane domains, and 4 conserved heme-binding histidines. NOXs have cell-specific distributions and play a role in many redox-sensitive physiological functions, including host defense, inflammation, cellular signaling, and cell growth [36]. It has been known since the discovery of the first member of the family, NOX2 (originally named gp91phox), that the NOXs play a role in the innate immune response to infections.

In phagocytic cells (neutrophils, macrophages, and microglia), the flavocytochrome b558, a heterodimer composed of NOX2 and the small protein p22phox, is responsible for the respiratory burst, which is triggered upon recognition of pathogens and results in the release of O_2_^•−^ into the phagocytic vacuole. O_2_^•−^ is rapidly converted to hydrogen peroxide (H_2_O_2_) and then to hydroxyl radical (OH^•^), via the Haber-Weiss reaction, and hypochlorous acid [36]. The cooperation of these ROS with granule-derived proteases and modification of the pH is essential for the killing of viruses and bacteria. Activation of the membrane-bound flavocytochrome b558 requires the association with proteins of cytosolic origin, namely p47phox, p67phox, p40phox and a small GTP protein (either Rac1 or Rac2), which promote the transition of flavocytochrome b558 from a resting to an active state for electron transport [37]. Formation of the active complex is the result of a series of phosphorylation-dependent protein-protein interactions that bring the activator subunit p67phox into contact with NOX2.

Although killing of virus in the vacuole was thought to be the unique role of NOX2, it has recently been reported to serve a critical function as a link between innate and adaptive immune responses [38,39]. Pathogens recognized by dendritic cells (DCs) are endocytosed and killed in the phagocytic vacuole, but an essential function of DCs is to ensure the correct proteolysis of pathogen-derived antigens in order to load them onto MHC class I or class II molecules to cross-prime and trigger cytotoxic activity of CD8+ T cells or activation and proliferation of naive CD4+ T cells, respectively. NOX2-derived O_2_^•−^ is associated with alkalinization of the phagosome through consumption of H^+^. This process results in the inactivation of proteases in the phagosome to prevent antigen degradation. It is recently reported that chronic hepatitis C virus (HCV) infection promotes the accumulation of CD33+ myeloid derived suppressor cells, resulting in ROS-mediated suppression of T cell responsiveness [40]. In this research, CD33+ mononuclear cells, co-cultured with HCV-infected hepatocytes, up-regulate the expression of p47^phox^, a component of the NOX2 and suppress autologous T cell responses. ROS might mediate the antigen presentation of phagocytes to T cells.

The high concentration of ROS produced in the vacuole can severely damage surrounding tissues through interaction with a large number of molecules, including proteins, lipids, and nucleic acids. The regulation of NOX-derived ROS at subtoxic concentrations by cellular signaling pathways is involved in innate immunity. Several homologues of NOX were found to act as signaling molecules downstream of TLRs. Interestingly, neutrophil-associated NOX2 was found to be essential for TLR4-mediated activation of NF-κB-dependent gene expression in neighboring lung endothelial cells after lipopolysaccharide (LPS) challenge [41]. This was the first demonstration that NOX2-derived ROS play a role in cell-to-cell communication and immune responses through the modulation of cell signaling. In contrast, it has recently been reported that an accelerated clearance of influenza virus in the absence of NOX2 activity was observed in infected mice [42]. In this study, *in vivo* administration of the NOX2 inhibitor apocynin significantly suppressed viral titer, airways inflammation and inflammatory cell superoxide production following infection. Inhibition of NOX2 expression in human airway epithelial cells also interfered with NF-κB-dependent innate immune responses following infection with respiratory syncytial virus (RSV) and Sendai virus infections [43]. NF-κB is a key transcription factor responsible for the regulation of cytokine and chemokine gene expression. In this research, it is revealed that NOX2 is an essential regulator of RSV and Sendai virus induced NF-κB activation acting upstream of both the phosphorylation of IκBα at Ser^32^ and of p65 at Ser^536^ in human bronchial epithelial cells. It is reported that ROS production via NOX2 might contribute to malignant transformation and tumor progression [44]. Epstein-Barr virus (EBV) nuclear antigen (EBNA)-1 from EBV induces chromosome aberrations engagement of the DNA damage response. EBNA-1 activates NOX2 and ROS production in EBNA-1 expressed cells. ROS from NOX2 might associate not only with innate immunity but also with tumor progression. These data suggest that phagocyte-associated NOX-derived ROS production may enable viruses to circumvent innate antiviral defenses. EMCV infection of the CNS is presumed to similarly link to innate immunity in microglia and lead to massive ROS production through NOX2. However, the role played by the NOX2-derived ROS in innate immunity has not yet been fully elucidated.

## 5. Cellular Prion Proteins Have a Neuroprotective Effect against Oxidative Injury Associated with EMCV Infection in the CNS

Taniuchi *et al.* reported that cellular prion protein (PrP^C^) has a protective function against EMCV infection *in vivo* [45]. PrP^C^ may be a key protein for protection against neuronal ROS damage arising from EMCV infection in the CNS. PrP^C^, a cell surface glycoprotein, is expressed mainly in neurons and also in glial cells, and it is an important factor in neurodegenerative prion diseases including bovine spongiform encephalopathy (BSE), scrapie, and Creutzufeldt-Jakob disease (CJD) [46]. Kuwahara *et al.* reported that PrP^C^-deficient neuronal cells die via apoptosis in serum-free medium, which indicates that PrP^C^ protects against oxidative stress under conditions of serum deprivation [47]. Sakudo *et al.* revealed that PrP^C^ has superoxide dismutase activity and protects neurons from oxidative stress [48]. In another report, infection of wild-type mice and PrP^C^-deficient mice with EMCV-B via the intracranial route caused encephalitis and induced neuronal cell apoptosis in vivo. There was no difference in virus titration levels between PrP^C^ gene-deficient (*Prnp*^−/−^*)* and wild-type *(Prnp^+/+^)* mice, but the degree of CA1 hippocampal pyramidal cells apoptosis was greater in *Prnp*^−/−^ than in *Prnp^+/+^* mice. In the CA1 region, there was greater microglia infiltration in *Prnp*^−/−^ than *Prnp^+/+^* mice [45]. These results suggest that PrP^C^ has antioxidant effects against ROS generated by microglia cells subsequent to virus infection. Kishimoto *et al.* reported that antioxidants reduce the severity of virus infection, indicating that ROS can promote EMCV infection [49]. This study investigated the antioxidant effect of coenzyme Q10 (CoQ10) against oxidative injury and DNA damage in mice infected with EMCV by the intraperitoneal route. The survival rate was significantly higher in CoQ10-treated versus control mice. Upregulation of thioredoxin due to DNA damage, which is induced by inflammatory stimuli triggered by the virus, was suppressed by CoQ10 treatment. Thus, it appears that PrP^C^ plays an important protective role against EMCV infection in the CNS.

## 6. Conclusions

**Figure 1:**
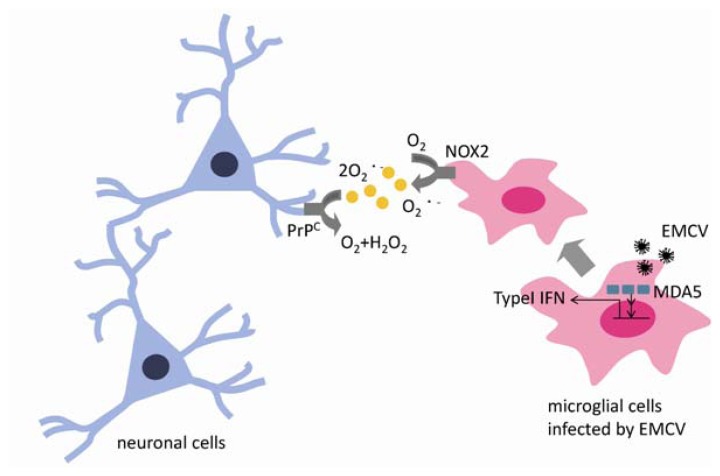
Encephalomyocarditis virus (EMCV) infection in the brain is recognized by microglia cells, which serve immunological and host-defense functions in the central nervous systems mediated by RIG and MDA. Activated microglia produce cytokines such as type 1 interferon and reactive oxygen spices (ROS) to clear the virus. ROS is produced mainly via NADPH oxidase 2, and overproduction of ROS is toxic to neurons. ROS-mediated injury to neurons is reduced by cellular prion proteins (PrP^C^) which has antioxidant effects and is neuroprotective against EMCV infection in the brain.

Many of the mechanisms involved in inflammation of the brain have yet to be clarified. The EMCV-B model is a convenient system for studying inflammation and neurotoxicity in the brain. ROS contributes to the activation of innate immunity as an antivirus system and to clear EMCV infection, but massive produced ROS by virus infection is very toxicity in each tissue. This review examined the mechanisms by which ROS produced during EMCV infection of the CNS trigger neuronal death, and the mechanisms by which PrP^C^ protects neurons from oxidative damage generated by EMCV infection (Figure 1). Regulation of inflammation and innate immunity in the brain is important for potential treatment for not only the virus infection but also neurodegenerative diseases including Alzheimer’s disease and Parkinson’s disease, because the inflammation is closely linked to neurodegenerative diseases. Further studies of antioxidants using the EMCV-B virus may help to advance treatment.
